# A screen of chromatin-targeting compounds identifies TAF1 as a novel regulator of HIV latency

**DOI:** 10.1128/mbio.01183-26

**Published:** 2026-06-15

**Authors:** Samuel D. Burgos, Airlie M. Ward, Manickam Ashokkumar, Kimberly P. Enders, Lindsey I. James, David M. Margolis, Edward P. Browne

**Affiliations:** 1 Department of Microbiology and Immunology, University of North Carolina School of Medicine 6797, Chapel Hill, North Carolina, USA; 2 Department of Medicine, University of North Carolina at Chapel Hill 214908, Chapel Hill, North Carolina, USA; 3 Department of Biostatistics, University of North Carolina Gillings School of Global Public Health, University of North Carolina at Chapel Hill 248512, Chapel Hill, North Carolina, USA; 4 Center for Integrative Chemical Biology and Drug Discovery, Division of Chemical Biology and Medicinal Chemistry, UNC Eshelman School of Pharmacy, University of North Carolina at Chapel Hill 2331, Chapel Hill, North Carolina, USA; 5 UNC Chapel Hill HIV Cure Center, University of North Carolina at Chapel Hill 2331, Chapel Hill, North Carolina, USA; University of California Davis School of Medicine, Davis, California, USA

**Keywords:** human immunodeficiency virus, transcription factors, HIV latency, latency reversing agents, Epigenetics, drug screening

## Abstract

Antiretroviral therapy suppresses HIV replication but fails to eliminate the virus due to the persistence of a transcriptionally silent reservoir, which remains the primary barrier to a cure. HIV latency is maintained through chromatin-mediated repression, making epigenetic regulators attractive therapeutic targets. To identify new modulators of latency, we screened a focused library of 84 chromatin-targeting small molecules. This screen identified BAY-299, a bromodomain inhibitor selective for TAF1 and BRD1, as a latency-modulating compound. BAY-299 reactivated HIV expression and enhanced the efficacy of established latency-reversing agents, including vorinostat, prostratin, and iBET-151, in cell line models. CRISPR/Cas9-mediated knockout experiments demonstrated that TAF1, but not BRD1, is essential for maintaining HIV latency and that TAF1 depletion selectively increases HIV transcription with minimal effects on host gene expression. Dual knockout of TAF1 and Tat revealed that reactivation of HIV in the absence of TAF1 is partially Tat-dependent. Cleavage Under Targets and Release Using Nuclease analysis further showed that TAF1 depletion increased histone acetylation at the viral promoter and across the HIV gene body, suggesting a chromatin-based mechanism. These findings identify TAF1 as a novel regulator of HIV latency and demonstrate the utility of targeted chemical screening to uncover therapeutic vulnerabilities within the latent reservoir.

**IMPORTANCE:**

HIV remains incurable due to the persistence of a transcriptionally silent reservoir in infected cells that is not eliminated by antiretroviral therapy. This transcriptionally silent state, known as latency, is controlled by host cell factors that regulate access to the viral genome. In this study, we identified the host protein TAF1 as a key regulator that maintains HIV in a latent state in cell line models of latency. Using both genetic and chemical approaches, we demonstrate that reducing TAF1 levels selectively increases HIV gene expression without broadly disrupting host gene transcription. These findings highlight a previously unrecognized mechanism of HIV latency control and identify TAF1 as a potential therapeutic target for HIV. Understanding how host chromatin regulators contribute to latency is essential for developing strategies that aim to eliminate the persistent HIV reservoir.

## INTRODUCTION

Antiretroviral therapy (ART) has transformed the management of HIV, improving quality of life and extending lifespans for people living with HIV (1–3). However, ART is not curative and fails to eliminate a rare but stable reservoir of latently infected cells harboring integrated, transcriptionally silent proviruses (4). These latently infected cells can sporadically reactivate, leading to rebound of viremia if treatment is interrupted (5). Thus, eliminating the latent reservoir remains a key objective in efforts to achieve a functional or sterilizing HIV cure.

To overcome HIV latency, considerable effort has been directed toward developing latency-reversing agents (LRAs) that reactivate latent proviruses, thereby either triggering cytopathic effects or enabling immune-mediated clearance of infected cells (6–13). LRAs such as histone deacetylase (HDAC) inhibitors and inhibitors of apoptosis proteins have shown promise in preclinical models by promoting HIV transcription (14). These compounds function by disrupting proviral silencing. However, most LRAs exhibit limited potency and breadth of reactivation, underscoring the need for a deeper understanding of latency mechanisms to inform alternative or combinatorial therapeutic strategies.

The persistence of the HIV reservoir is driven by host chromatin regulation, particularly covalent histone modifications, such as acetylation and methylation, which shape proviral chromatin structure. Histones H3 and H4 possess modifiable tails that are targeted by protein complexes that read, write, or erase these marks (15, 16). Once integrated, HIV is governed by the same chromatin-based mechanisms that regulate host transcription, including histone modifications and nucleosome remodeling (17–19). These processes can restrict access to the transcriptional machinery, maintaining latency and suppressing reactivation. Given the complexity of these pathways, chromatin regulation offers multiple potential therapeutic targets. Although some LRAs disrupt silencing mechanisms, most fail to reactivate the bulk of the latent reservoir (20, 21), highlighting the need to identify additional compounds that engage distinct regulatory pathways and to test possible combinations with current LRAs.

To identify novel chromatin-associated factors that regulate HIV latency, we screened a library of 84 small molecules targeting enzymes involved in writing or erasing histone modifications, including bromodomain proteins (BRDs), lysine demethylases (KDMs), histone acetyltransferases (HATs), deacetylases (HDACs), methyltransferases (HMTs), and methyl-lysine readers (Kme readers). The screen identified latency-modulating compounds across several epigenetic classes, with particularly strong activity among BRD and HDAC inhibitors. One notable hit was BAY-299, a bromodomain inhibitor that is selective for TAF1 and BRD1 (22), which enhanced HIV expression and sensitized latently infected cells to canonical LRAs in cell lines. CRISPR/Cas9 knockout experiments confirmed that TAF1, not BRD1, is required to maintain HIV latency in cell lines. These findings demonstrate the power of targeted chemical screens to reveal novel regulatory pathways and therapeutic vulnerabilities within the latent HIV reservoir.

## RESULTS

### A chemical screen identifies novel compounds affecting HIV latency

**Fig 1:**
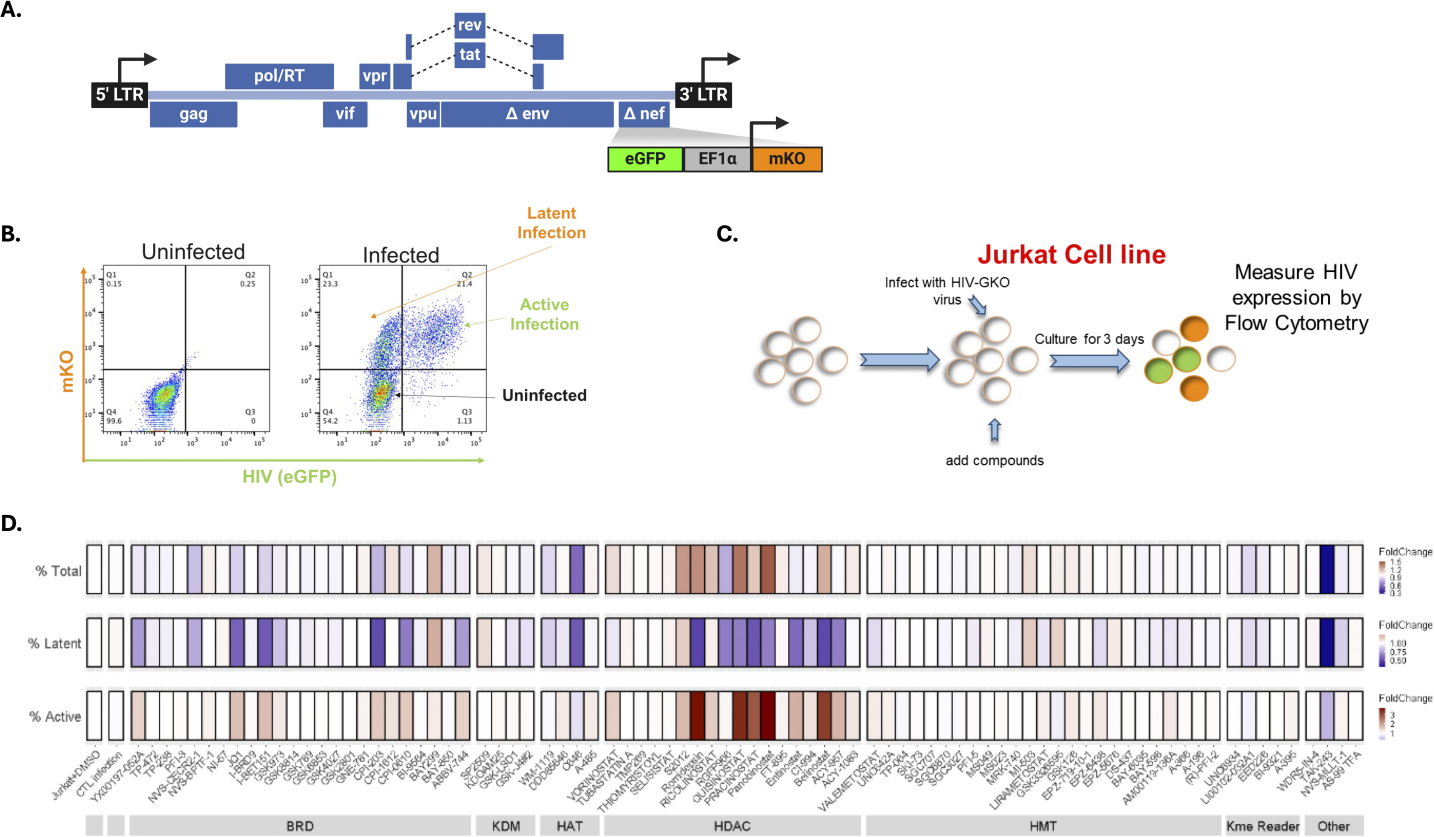
*Chemical screen identifies novel chemical compounds affecting HIV latency*. (**A**) The HIV-GKO virus map illustrating the expressed viral genes and the location and identity of the fluorophore cassettes, adapted from Battivelli et al. (23). (**B**) Example of flow cytometry of HIV-GKO-infected Jurkat cells, highlighting latent and active infection based on the viral promoter driving GFP expression and the constitutive promoter EF1α driving mKO expression. (**C**) Schematic representation of small molecule compound screening in Jurkat cell line model of HIV latency. (**D**) A multivariable heatmap illustrates changes relative to the DMSO control for total infection percentage, latent infection percentage, and active infection percentage. Data shown are the average of four replicates.

To identify novel compounds that affect HIV latency, we screened an 84-compound library targeting proteins that bind, add, or remove post-translational histone modifications. The screen was conducted in Jurkat cells, a human CD4^+^ T-cell line, infected with the HIV-GKO strain (23). HIV-GKO is a dual-color reporter virus that maintains expression of most viral genes but includes a non-functional env gene and two fluorophores: enhanced green fluorescent protein (eGFP) under the HIV-1 5′ LTR promoter and mKO under the EF1α promoter within the *nef* gene (Fig. 1A). This virus thus enables quantification of both active (eGFP^+^/mKO^+^) and latent (eGFP^−^/mKO^+^) infection within an infected population of cells (Fig. 1B).

HIV-GKO-infected Jurkat cells were treated with each compound at 1 µM in a 96-well plate format to enable high-throughput screening (Fig. 1C). After 3 days, cells were analyzed by flow cytometry to assess changes in infection and viral expression. Live cells were gated using a viability dye, then assessed for mKO expression to determine total infection (% total infection). mKO-positive (mKO^+^) cells were further gated based on eGFP expression to define latently (eGFP⁻/mKO^+^) and actively (eGFP^+^/mKO^+^) infected cells. Fold changes for each population were calculated relative to vehicle (dimethyl sulfoxide [DMSO]) controls (Fig. 1D; Table 1; Table S1). Overall, 32 compounds caused a change in frequency for either the actively infected or latently infected populations. None of the compounds screened significantly altered total infection (all false discovery rate [FDR] > 0.05). An increase in the percentage of latently infected cells was observed for five compounds under these conditions (FDR < 0.05): BAY-299, SP2509, S2012, GSK3326595, and MI503. Conversely, 20 compounds—ABB744, CPI-0610, CPI-203, GSK973, iBET151, JQ1, NVS-CECR2-1, iBET-762, C646, ACY957, belinostat, CI-994, pracinostat, quisinostat, RGFP966, panobinostat, entinostat, romidepsin, NVS-MLLT1, and TAK243—produced a significant decrease in the latent cell population (Table 1; Table S1). Finally, 24 compounds significantly increased the percentage of actively infected cells (FDR < 0.05), including ABBV-744, BAY-299, CPI-0610, CPI-1612, CPI-203, GSK973, I-BET151, JQ1, iBET-762, A-485, ACY-957, belinostat, CI-994, panobinostat, entinostat, pracinostat, quisinostat, ricolinostat, RGFP966, romidepsin, S2012, vorinostat, UNC1999a, EPZ-6438, and UNC642A, whereas only TAK-243 significantly reduced active infection (Table 1). The results of this screen approach demonstrate the approaches’ capability to identify novel small molecules that influence HIV expression and latency.

**TABLE 1:** Summary of compounds that modulate HIV infection and expression^*a*^

Compound	Class	Active FC	Latent FC
ABBV-744	BETi	**1.50**	**−1.38**
BAY-299	BETi	**1.25**	**1.18**
CPI-0610	BETi	**1.50**	**−1.57**
CPI-1612	BETi	**1.25**	1.00
CPI-203	BETi	**1.50**	**−2.20**
GSK973	BETi	**1.25**	**−1.22**
iBET151	BETi	**1.75**	**−1.83**
JQ1	BETi	**1.75**	**−1.83**
NVS-CECR2-1	BETi	1.00	**−1.38**
iBET762	BETi	**1.50**	**−1.38**
SP2509	KDMi	1.00	**1.09**
A-485	HATi	**1.25**	−1.10
C646	HATi	−1.33	**−1.83**
ACY-957	HDACi	**1.75**	**−1.57**
Belinostat	HDACi	**3.50**	**−2.20**
CI-994	HDACi	**1.25**	**−1.22**
Pracinostat	HDACi	**3.00**	**−1.83**
Quisinostat	HDACi	**3.50**	**−1.57**
RGFP966	HDACi	1.00	**−1.57**
Ricilinostat	HDACi	**1.50**	1.00
Panobinostat	HDACi	**3.75**	**−1.57**
Entinostat	HDACi	**2.00**	**−1.83**
S2012	HDACi	**2.00**	**1.09**
Romidepsin	HDACi	**3.75**	**−2.20**
Vorinostat	HDACi	**1.50**	−1.10
UNC1999A	HMTi	**1.25**	−1.10
EPZ-6438	HMTi	**1.25**	−1.10
UNC642A	HMTi	**1.25**	−1.10
GSK3326595	HMTi	−1.33	**1.09**
MI-503	HMTi	1.00	**1.09**
NVS-MLLT-1	Other	1.00	**−1.22**
TAK-243	Other	**−4.00**	**−3.67**

^*a*^ Compounds that affected the abundance of cells in either of the infected population subsets (latent infection, active infection) are shown. Numbers represent fold change. Bold indicates statistical significance (Pval_adj _< 0.05). Active FC, fold change in actively infected population; Latent FC, fold change in latently infected population; BETi, bromodomain and extraterminal domain inhibitor; KDMi, lysine demethylase inhibitor; HATi, histone acetyltransferase inhibitor; HDACi, histone deacetylase inhibitor; HMTi, histone methyltransferase inhibitor.

### BAY-299 affects latency reversal in a cell line model of HIV latency

**Fig 2:**
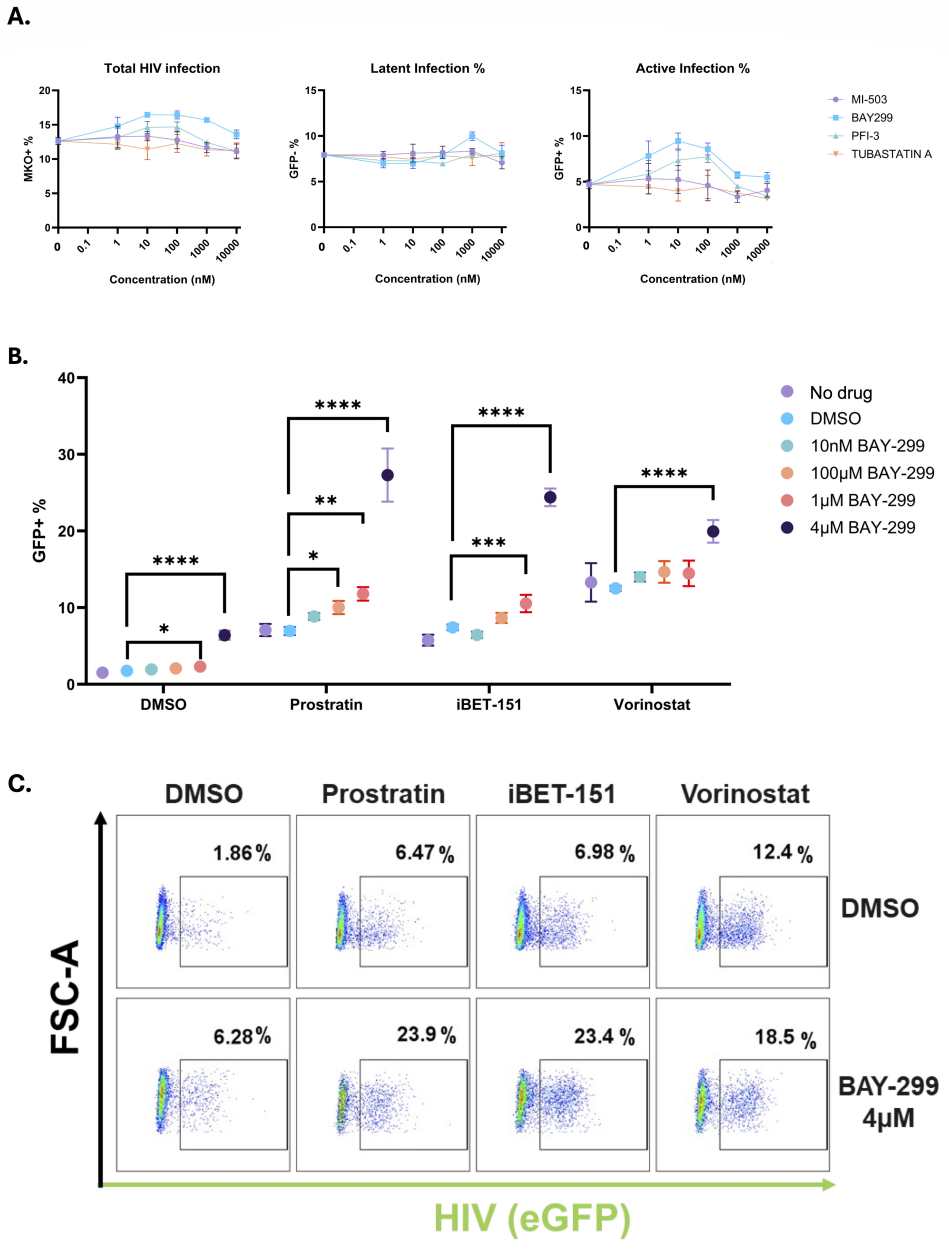
*BAY-299 affects HIV gene expression and enhances latency reversal in cell lines*. (**A**) Dose-response curves for selected compounds: MI-503, BAY-299, PFI-3, and Tubastatin A, showing the effects on total HIV infection (mKO^+^), latent infection (mKO^+^, GFP^−^), and active infection (mKO^+^, GFP^+^) percentages. Jurkat cells were infected with HIV-GKO and exposed to each compound for 72 h before flow cytometry. DMSO control is plotted as a single dot on the plot. Datapoints represent the average of technical triplicates. (**B**) Dose-response curve for BAY-299 in 2D10 cells ranging from 10 nM to 4 µM, with and without the addition of LRAs prostratin (125 nM), iBET-151 (125 nM), and vorinostat (250 nM). Measurements are an average of four technical replicates. *P*-value: ****P < 0.0001, ***P < 0.001, **P < 0.01, *P < 0.05. (**C**) Flow cytometry showing the changes in GFP expression of 2D10 cells when treated with LRAs alone and LRAs in conjunction with BAY-299 at 4 µM.

In addition to known LRAs, the screen identified novel compounds, including several from classes not previously studied in the context of HIV. We selected four for further analysis: one that increased total infection and latent infection (BAY-299, a TAF1/BRD1 inhibitor), one that increased latent infection (MI-503, a Menin-MLL inhibitor), and two that did not alter HIV infection in the screen at 1 μM but which had mechanisms of action that have not been previously studied for HIV: PFI-3 (a SMARCA2/4 inhibitor) and Tubastatin A (an HDAC6 inhibitor). To validate their activity, we performed dose-response analyses in HIV-GKO-infected Jurkat cells across concentrations from 1 nM to 10 μM, measuring infection profiles by flow cytometry at 3 days post-infection. BAY-299 and PFI-3 both increased total infection at intermediate doses (10–100 nM) (Fig. 2A). At 1 μM, BAY-299 also modestly increased latent infection, consistent with initial screen results, while other compounds showed no significant change from DMSO controls. Notably, active infection (eGFP^+^/mKO^+^) was significantly elevated by BAY-299 and PFI-3 at 10–100 nM. BAY-299 induced the strongest activation within this range but showed reduced effect at higher doses (1–10 µM). Similarly, PFI-3 activity peaked between 10 and 100 nM. These results suggest that, while 1 μM was used in the initial screen, both compounds exhibit peak activity around 100 nM. BAY-299 thus shows a concentration-dependent dual effect, enhancing activation at lower doses while modestly increasing latency at higher concentrations.

We next tested BAY-299 in 2D10 cells, a well-established model of HIV latency containing a quiescent provirus that encodes Tat, Env, Vpu, Rev, and eGFP under the 5′ LTR promoter (24). Cells were treated with BAY-299 at concentrations from 10 nM to 4 µM for 24 h. Unlike the HIV-GKO model, where peak activation occurred at 10–100 nM, BAY-299 induced modest reactivation in 2D10 cells only at 1 and 4 µM (Fig. 2B). A broader dose curve (100 nM to 10 µM) indicated significant latency reversal activity for BAY-299 in 2D10 cells at 4 and 10 µM concentrations (Fig. S1A) while also causing a modest reduction in cell viability at both concentrations (Fig. S1B). We also tested whether BAY-299 could enhance the activity of other LRAs: prostratin (125 nM), iBET-151 (125 nM), and vorinostat (250 nM), by co-stimulating 2D10 cells and measuring HIV expression by flow cytometry for eGFP at 24 h post-stimulation. Notably, combining BAY-299 at 1 or 4 µM with any of these LRAs significantly increased GFP expression compared to LRA treatment alone (Fig. 2B and C). This enhancement was strongest in combination with prostratin or iBET-151, producing a threefold increase relative to DMSO controls. Bliss independence analysis indicated a modest synergistic interaction between BAY-299 and both prostratin and iBET-151 at higher concentrations, but not with vorinostat (Table S2). We also tested the ability of BAY-299 to induce HIV expression in a primary cell model of HIV latency, both alone or in combination with LRAs, and found that BAY-299 inhibited HIV expression and latency reversal in primary cells, suggesting that its latency reversing activity is specific to cell line models of HIV latency (Fig. S2), and that TAF1 may play a distinct role in primary cell latency models. Overall, these results show that BAY-299 has modest latency-reversing activity in and can significantly potentiate the effects of diverse LRAs in a cell line model of latency but inhibits HIV reactivation in primary CD4 T cells.

### TAF1 is required for HIV latency in cell line models

**Fig 3:**
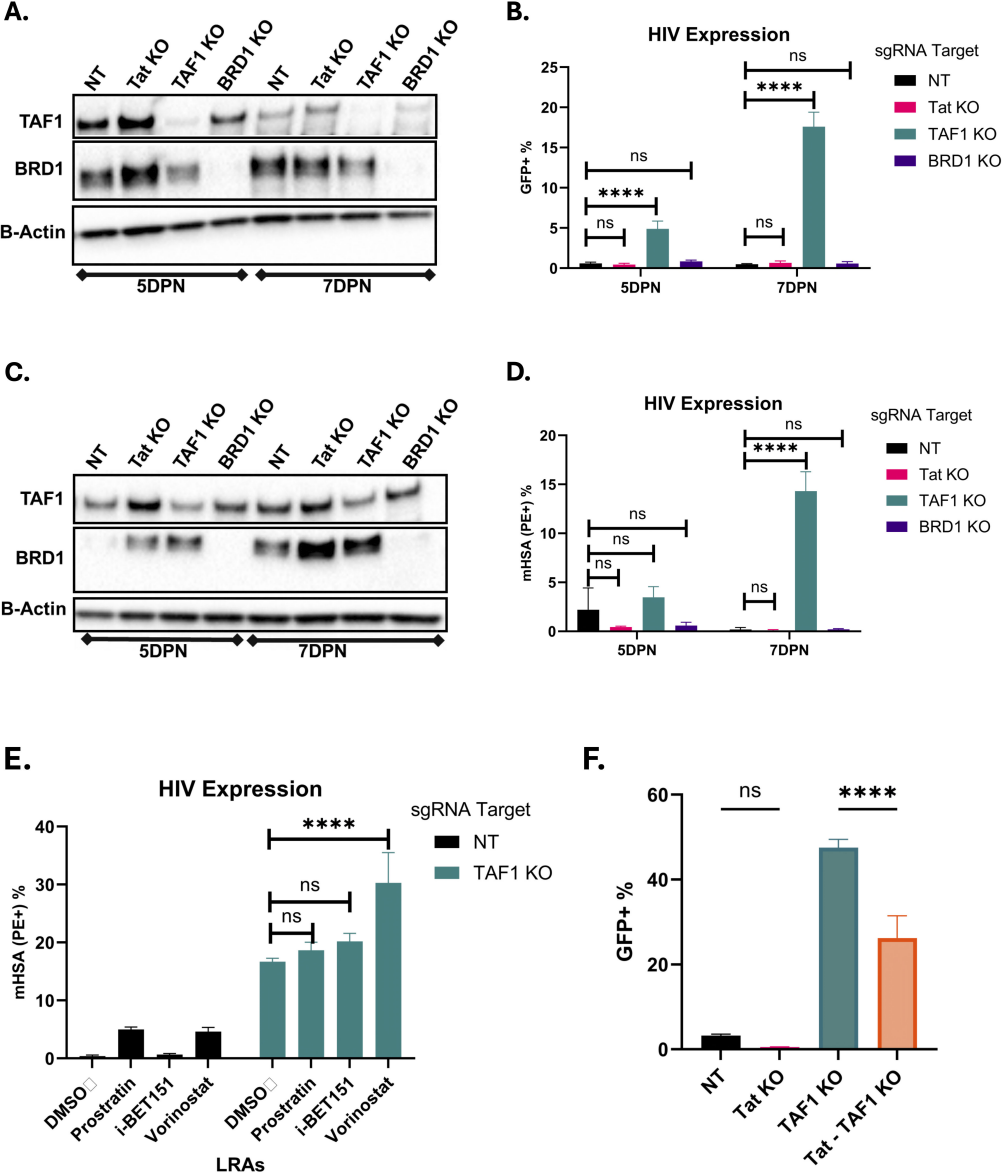
*TAF1 depletion leads to Tat-dependent HIV reactivation*. (**A**) Western blot of 2D10 cells following CRISPR/Cas9 nucleofection targeting TAF1 and BRD1 at 5 and 7 days post-nucleofection (DPN). Non-targeting gRNA (NT) represents a negative control. (**B**) Bar graph showing the changes in average eGFP expression determined by flow cytometry from three replicates in 2D10 cells, comparing the effects of TAF1 and BRD1 depletion. *P*-value: ****P < 0.0001. (**C**) Western blot of CRISPR/Cas9-depleted N6 cells targeting Tat, TAF1, and BRD1 at 5 and 7 DPN. (**D**) Bar graph showing the changes in average eGFP expression determined by flow cytometry from three replicates of N6 cells, comparing the effects of Tat, TAF1, and BRD1 depletion. (**E**) Bar graph showing the effect of 24-h treatment of different LRAs (prostratin 250 nM, iBET151 250 nM, and vorinostat 500 nM) on the expression of HIV (measured by mHSA/PE^+^) in the N6 cell line with or without TAF1 depletion at 7 DPN. *P*-value: ****P < 0.0001. (**F**) Bar graph showing the effect of TAF1 knockout, Tat knockout, and TAF1/Tat double knockout on the percentage of eGFP-positive cells in the 2D10 cell line at 7 DPN. *P*-value: ****P < 0.0001.

BAY-299 was initially developed as a selective inhibitor for Bromodomain and PHD Finger-Containing Protein 2 (BRPF2/BRD1) (22). Although it lacks affinity for the related proteins BRPF1 and BRPF3, the same study reported potent binding to the second bromodomain of TAF1. We hypothesized that BAY-299 affects HIV latency by targeting either BRD1 or TAF1. To test this, we nucleofected CRISPR-Cas9 ribonucleoprotein (RNP) complexes targeting TAF1, BRD1, or a non-targeting control into 2D10 cells. Western blot confirmed robust protein depletion by 5 days post-nucleofection (DPN) (Fig. 3A). HIV expression was assessed by measuring eGFP by flow cytometry at 5 and 7 DPN. Notably, TAF1 depletion, but not BRD1 depletion, significantly increased viral eGFP expression (Fig. 3B), suggesting that TAF1 is important for maintaining HIV latency in 2D10 cells.

To further validate this result, we tested TAF1 and BRD1 depletion in a second latency model, the N6 cell line. N6 cells harbor a full-length HIV provirus and encode mouse heat-stable antigen (mHSA) in the *nef* open reading frame, allowing quantification of HIV expression by flow cytometry for mHSA (25). Compared to 2D10 cells, TAF1 depletion in N6 cells was incomplete, while BRD1 knockdown was more pronounced by 5 and 7 DPN (Fig. 3C). Despite this, partial TAF1 depletion led to a significant increase in HIV expression, as measured by mHSA staining at day 7 (Fig. 3D). These data support a role for TAF1, but not BRD1, in repressing HIV across multiple cell line latency models.

We next examined whether TAF1 or BRD1 depletion could enhance HIV reactivation in response to latency-reversing agents (LRAs). N6 cells depleted for TAF1 or BRD1 were treated with suboptimal doses of vorinostat, prostratin, or iBET-151. Among these, only vorinostat exhibited an additive effect with TAF1 depletion, while the others showed no additional increase in HIV expression (Fig. 3E). This suggests that TAF1 loss can independently reverse latency and selectively enhance the activity of certain LRAs.

To further investigate the mechanism by which TAF1 depletion increases HIV expression, we performed a dual knockout of TAF1 and Tat, the HIV-encoded transcriptional activator. Prior studies have shown that even without multiple TAFs, including TAF1, components of the pre-initiation complex can still bind the HIV promoter, with Tat potentially compensating for loss of TAFs (26). In 2D10 cells, we nucleofected RNPs targeting TAF1, Tat, or both. Consistent with prior results, TAF1 depletion alone elevated HIV expression (Fig. 3F). Tat depletion alone caused a reduction in the baseline level of background HIV expression, consistent with Tat’s known role as a positive regulator of HIV expression, although this change was not statistically significant. However, combined targeting of TAF1 and Tat significantly reduced the increase observed with TAF1 knockout, although HIV expression remained above that of the non-targeting (NT) or Tat-only groups. Attempts to verify Tat expression and depletion by Western blot were unsuccessful, although the reduced expression of HIV in both baseline and TAF1-targeting conditions with Tat knockout suggests that effective targeting of the Tat gene was occurring. These results suggest that Tat contributes to the transcriptional enhancement observed following TAF1 depletion. We also examined whether BAY-299 exhibited LRA activity in the absence of TAF1 by stimulating TAF1-depleted 2D10 cells or control cells with BAY-299 and found that, interestingly, BAY-299 largely retained its modest LRA activity at high doses in the absence of TAF1, suggesting that high-dose BAY-299 likely has additional cellular targets that can mediate its effect on HIV transcription in the absence of TAF1 (Fig. S3).

### TAF1 depletion in 2D10 cells results in the preferential upregulation of HIV transcription

**Fig 4:**
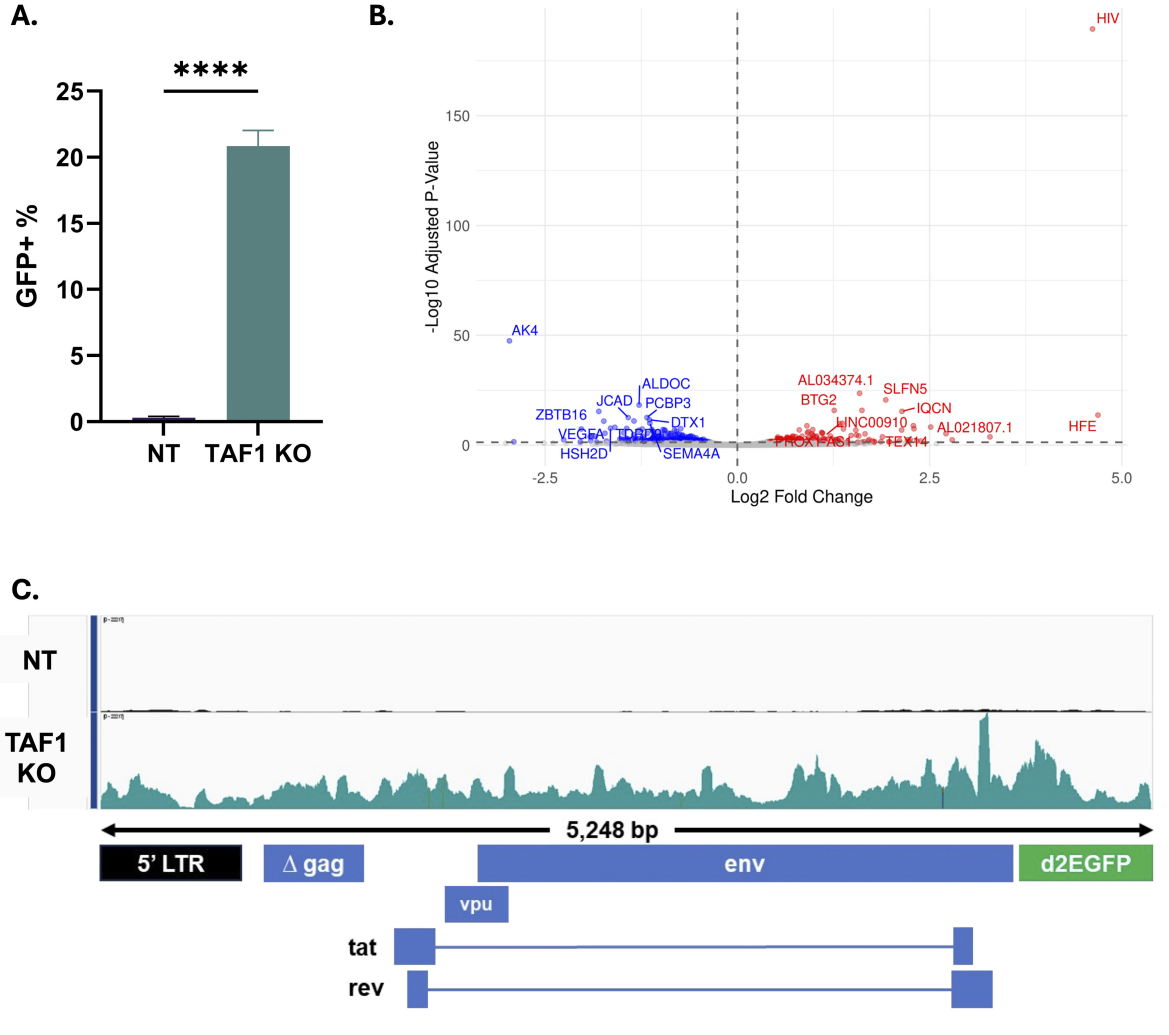
*TAF1 depletion in 2D10 cells results in a preferential increase in HIV transcripts*. 2D10 cells were nucleofected with either NT or TAF1-targeting ribonucleoprotein (RNP) complexes, and, at 7 days post-nucleofection, cells were harvested for flow cytometry and RNA isolation for RNA sequencing. (**A**) Bar graph showing that TAF1 depletion in 2D10 cells leads to a significant increase in eGFP^+^ cells at 7 days post-nucleofection, indicating elevated HIV expression in the same experimental context used for transcriptomic profiling. *P*-value: ****P < 0.0001. (**B**) Volcano plot highlighting significantly upregulated (red) and downregulated (blue) genes in TAF1-depleted cells versus non-targeting controls. Colored points represent significant genes with adjusted *P*-values (log pval_adj_) less than 0.05, while gray points represent genes that are not significantly changed. (**C**) RNA-seq coverage tracks of the HIV genome in 2D10 cells, comparing non-targeting CRISPR/Cas9-treated cells and TAF1-depleted cells. Data are derived from three independent biological replicates.

TAF1 has been reported to facilitate transcriptional initiation by recruiting the TFIID complex to acetylated histones at gene promoters (27, 28). We hypothesized that TAF1 depletion might directly affect HIV transcription or alter expression of host genes that regulate latency. To test this, we depleted TAF1 in 2D10 cells using CRISPR-Cas9 nucleofection and confirmed increased viral GFP expression by flow cytometry (Fig. 4A). RNA from TAF1-depleted and non-targeting control cells was then analyzed by bulk RNA-sequencing (RNA-seq). Principal component analysis of the transcriptional profiles showed consistent separation between TAF1-depleted and control cells, indicating a clear biological signal in responses to TAF1 knockout (Fig. S4). Differential expression analysis using DESeq2 identified 163 significantly upregulated and 354 significantly downregulated genes (pval_adj_ < 0.05) in TAF1-depleted cells (Fig. 4B). Notably, HIV was the most significantly upregulated transcript, suggesting that TAF1 plays a relatively selective role in repressing HIV expression in this model. The effect of TAF1 KO on HIV expression could be further observed when we examined RNA sequencing coverage across the HIV provirus, which was greatly increased in TAF1-depleted cells compared to NT control (Fig. 4C). Pathway enrichment analysis revealed no clear enrichment of functional pathways among differentially expressed genes, indicating that TAF1 depletion drives robust HIV reactivation with only modest effects on host gene transcription.

### TAF1 depletion regulates histone acetylation within the HIV provirus

**Fig 5:**
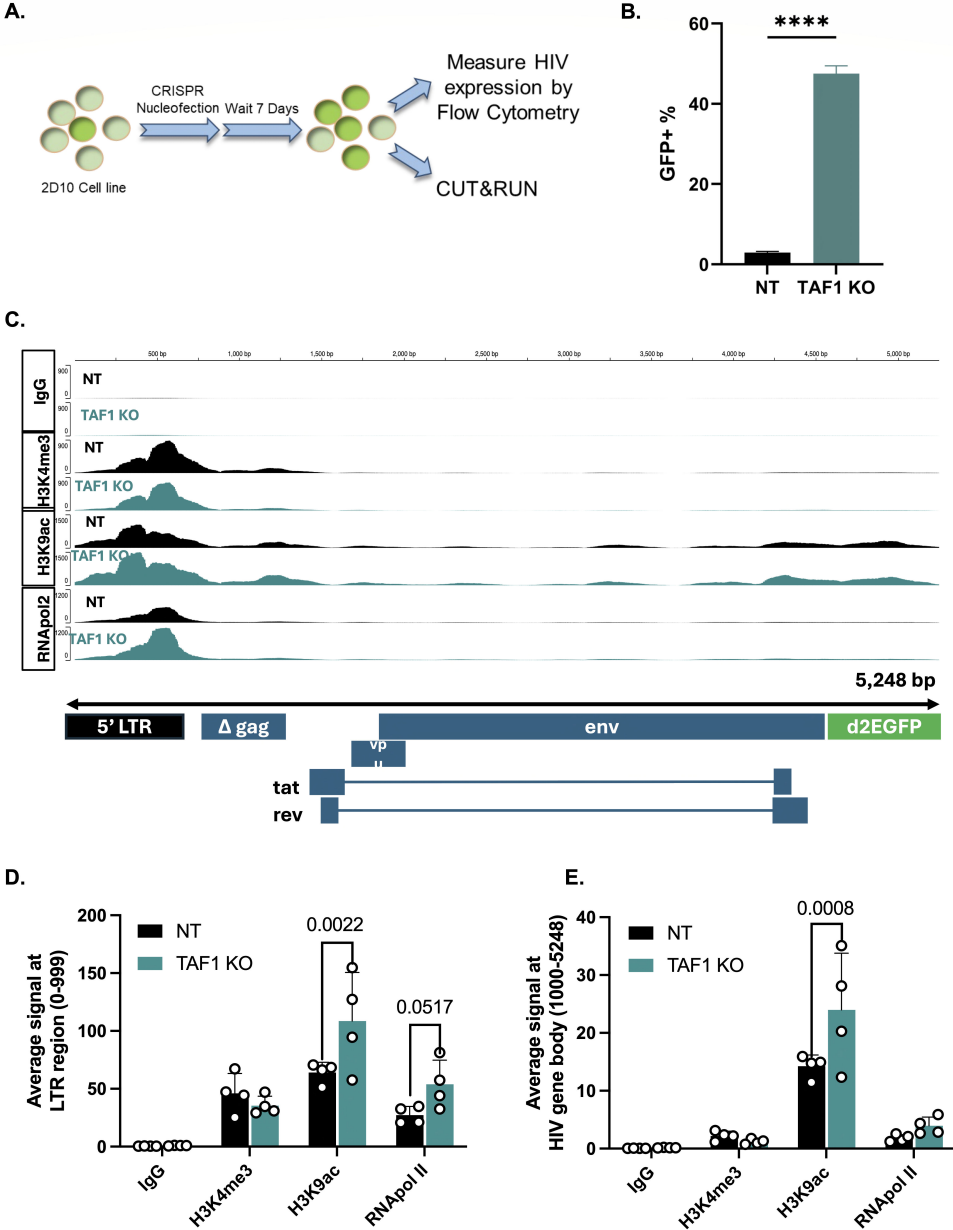
*TAF1 depletion in 2D10 cells increases histone acetylation within the HIV provirus*. (**A**) Schematic illustration of CUT&RUN experimental design. 2D10 cells were nucleofected with either NT or TAF1-targeting ribonucleoprotein (RNP) complexes. At 7 days post nucleofection (DPN), cells were analyzed for eGFP expression by flow cytometry and processed for CUT&RUN profiling. (**B**) Bar graph showing increase in eGFP^+^ cells following TAF1 depletion in 2D10 cells at 7 DPN, *P*-value: ****P < 0.0001. (**C**) CUT&RUN sequence coverage of the HIV genome. Tracks correspond to coverage for either IgG (negative control), H3K4me3, H3K9ac, or RNAPol II for NT or TAF1-depleted 2D10 cells. (**D**) Bar graph showing fold change read counts for IgG, H3K9ac, H3K4me3, and RNAPol II (Rbp1) abundance within the HIV LTR region (nts 0–1,000). (**E**) Bar graph showing fold change read counts for IgG, H3K9ac, H3K4me3, and RNAPol II (Rbp1) abundance across the HIV gene body (nts 1,000 to 5,248). Data are derived from four independent biological replicates. *P*-values for statistically significant differences (Kruskal-Wallis test, *P* < 0.05) are shown.

To investigate how TAF1 depletion enhances HIV transcription in 2D10 cells, we performed Cleavage Under Targets and Release Using Nuclease (CUT&RUN) to examine activating histone marks and RNAPol II at the HIV provirus. Cells were nucleofected with CRISPR/Cas9 RNPs targeting TAF1 or a non-targeting (NT) control (Fig. 5A). At 7 days post-nucleofection, HIV reactivation was confirmed by increased eGFP expression (Fig. 5B). CUT&RUN was conducted using antibodies against H3K4me3 and H3K9ac (activating histone marks), RNAPol II, and IgG control. As expected, we observed enrichment of H3K4me3, H3K9ac, and RNAPol II at the HIV LTR near the transcription start site, consistent with prior reports (29). TAF1-depleted cells showed a significant increase in H3K9ac abundance at the HIV LTR, while H3K4me3 remained largely unchanged (Fig. 5C and D). RNAPol II signal at the LTR also trended upward, although this did not reach statistical significance. Across the HIV gene body, we similarly observed increased H3K9ac signal in TAF1-depleted cells, whereas H3K4me3 and RNAPol II showed little change (Fig. 5E). Together, these data indicate that TAF1 depletion is associated with altered chromatin acetylation across the HIV provirus, particularly increased H3K9ac. These findings suggest that TAF1 represses HIV transcription in cell lines at least in part by limiting accumulation of activating chromatin marks at the provirus.

## DISCUSSION

The persistence of latently infected cells remains the primary barrier to achieving an HIV cure, as this reservoir evades immune surveillance and resists ART (30, 31). Despite significant advancements in LRAs, there is still limited efficacy in fully reactivating the latent reservoir, highlighting the need for novel therapeutic approaches (32). LRAs such as panobinostat and vorinostat (HDACi), JQ1 and iBET151 (BRD4i), and AZD5582 (inhibitor of apoptosis proteins) are well-studied compounds that have demonstrated the ability to increase HIV gene expression via different mechanisms. However, individually, they have not been able to broadly reactivate the latent reservoir (33).

In this study, we found that many of the compounds that increased the percentage of actively expressing HIV cells are members of the HDAC inhibitor (HDACi) family or bromodomain (BET) inhibitors that interfere with the binding of bromodomain-containing proteins to acetylated histones. This observation highlights the central importance of histone acetylation in HIV latency, consistent with previous reports (7, 10, 20, 34, 35). Several of these compounds have previously been identified as LRAs, including the HDACis romidepsin, panobinostat, and vorinostat, as well as the BETis iBET-151 and JQ1. This observation thus gives us confidence in our screen by validating that we were able to identify known LRAs. Compounds from the other categories in the library did not affect the percent active population, except for TAK-243, an inhibitor of ubiquitin-activating enzyme, which reduced the percent active population, highlighting a different avenue to affect HIV transcription.

From the overall set of biologically active compounds identified by this screen, we selected BAY-299 for further analysis. Subsequent analysis confirmed BAY-299’s ability to increase the percent total infection, percent latent, and percent active infection in Jurkat cell line models of HIV infection. Notably, when used in combination with different LRAs such as prostratin, iBET-151, and vorinostat, 1 or 4 μM BAY-299 further enhanced HIV transcription, demonstrating a possible combinatorial effect on HIV transcription. BAY-299 is a bromodomain inhibitor specifically designed to differentiate between bromodomain paralogs, with selectivity for BRD1 and TAF1 (22). Given that this compound influenced all measured parameters related to changes in HIV latency and transcription, we further investigated the possible mechanism by which BAY-299 affects HIV by selective depletion of its targets BRD1 or TAF1. Notably, depletion of TAF1 but not BRD1 caused a robust increase in HIV transcription in two cell lines that were latently infected with HIV (2D10 cells and N6 cells). Additionally, the changes in gene expression caused by TAF1 depletion appear to be relatively selective for HIV transcription, with minimal impact on host gene expression. This observation is particularly intriguing because TAF1 is a subunit of the TFIID complex, which plays an important role in transcriptional initiation as the rate-limiting step for RNA polymerase II binding to DNA (27). Given TAF1’s well-known role in facilitating transcriptional initiation, it is curious that TAF1 exhibits a specific HIV-repressing activity in these cells. Notably, previous *in vitro* studies have demonstrated that HIV transcription can occur in the absence of TAF subunits within the TFIID complex, including TAF1, suggesting the existence of compensatory mechanisms that enable transcription despite their depletion (26). It has been proposed that HIV maintains transcription under these conditions through a compensatory mechanism involving Tat, an essential viral protein for HIV transcription (26). This hypothesis aligns with our RNA-seq data that demonstrate a specific increase in HIV transcription in the absence of TAF1. Interestingly, TAF1-depleted 2D10 cells still exhibited responsiveness to high-dose BAY-299 with respect to latency reversal, suggesting that BAY-299 may have additional targets other than BRD1 and TAF1 that regulate HIV expression. It will be important to identify these unknown targets and clarify their interaction with HIV transcription machinery, including TAF1. We also found that, while BAY-299 can enhance HIV expression in cell line models of latency, it inhibited HIV reactivation in a primary CD4 T-cell model of latency, suggesting important differences between these models with respect to the role of BAY-299 targets in HIV expression.

Previous work has shown that TAF7 contains a domain that binds TAF1, enabling formation of the TFIID pre-initiation complex and simultaneously inhibiting the acetyltransferase activity of TAF1. This interaction is thought to function as a transcriptional checkpoint regulated by TAF7 (36). It was also demonstrated that HIV-1 Tat, the HIV-encoded transcriptional activator, contains a structurally similar domain to TAF7, allowing it to bind TAF1 in a comparable manner. This interaction has been proposed to downregulate the expression of TAF1-dependent host genes, such as MHC class I, potentially contributing to immune evasion by reducing antigen presentation (37). Based on these findings, we hypothesize that in HIV cell line models, depletion of TAF1 reduces the availability of TAF1 for Tat binding. As a result, Tat may be more available to drive transcription at the HIV promoter in the absence of TAF1, leading to the observed increase in HIV gene expression. This is supported by the robust increase in HIV-1 mRNA levels and increase in eGFP expression in TAF1-depleted cells.

Additionally, our CUT&RUN data reveal increased H3K9ac abundance at both the HIV LTR and across the HIV gene body in the absence of TAF1, consistent with a more transcriptionally permissive chromatin state at the provirus. While RNAPol II signal at the LTR trended upward, we did not observe a strong selective increase in RNAPol II occupancy across the HIV gene body. Taken together, these data are consistent with a model in which TAF1 depletion relieves Tat sequestration, allowing greater Tat availability to promote HIV transcription, while also being associated with increased activating chromatin marks across the provirus.

It remains unclear whether TAF1 is directly or indirectly associated with the HIV provirus in latently infected cells, and addressing this question will be important to establish the precise role of TAF1 in regulation of HIV expression. We also speculate that, in latently infected cell lines, TAF1 could be a key component of a stalled non-productive transcriptional complex that is recruited to the LTR promoter of latent proviruses, and TAF1 depletion releases this stalled complex, allowing reinitiation of productive transcription. The increased acetylation of histones within the HIV provirus after TAF1 depletion also suggests a connection between TAF1 and histone acetylation machinery. It is possible, for example, that TAF1 contributes to recruiting histone deacetylases (HDACs) to latent proviruses, thereby mediating viral silencing through loss of H3K9Ac marks. Consistent with this observation, we observed an increased level of H3K9Ac modification at the HIV provirus after TAF1 depletion.

Our results from this screen should be considered in light of several limitations and caveats. While this screen was effective in identifying novel compounds that influence HIV latency, it was performed using immortalized cell lines as the model for measuring changes in HIV transcription, which exhibit several differences from primary CD4 T cells, the primary location of the clinical reservoir (38, 39). Consequently, it is unclear that the findings from this screen will translate to primary cells or *in vivo* models of HIV latency. Indeed, we observed that BAY-299 was effective at promoting HIV expression in cell lines but inhibited HIV expression in primary CD4 T cells. The library used in this screen consisted of 84 distinct small molecules that target various chromatin functions critical to transcription. To standardize the approach, however, we selected a single concentration of 1 µM, a dose low enough to minimize toxicity yet sufficient to be effective for most compounds. However, due to the variability in efficacy, selectivity profiles, and toxicity of these compounds, the optimal dose for many compounds likely differs from the concentration used in the screen. Nevertheless, at this concentration, we observed notable effects in response to several compounds. Future screening could potentially examine the compound set across multiple concentrations in order to more thoroughly evaluate the biological activity of the molecules. Another variable to consider is the timing of compound treatment. In this screen, compounds were added on the same day as infection to capture changes in total infection, latent infection, and active infection. However, as the HIV integration complex takes 24–48 h to fully integrate into the host genome, the timing of compound addition could influence the results.

Despite these limitations, this screen serves as a robust template for identifying novel epigenetic mechanisms and pathways that can influence HIV transcription. Through this approach, we identified BAY-299, a compound that affects HIV latency by targeting TAF1 and modulating HIV transcription.

## MATERIALS AND METHODS

### Cell line models of HIV latency

The 2D10 and Jurkat-N6 latently infected cell lines (25, 40), along with parental Jurkat cells, were maintained in RPMI-1640 medium (Gibco, Thermo Fisher), supplemented with 10% fetal bovine serum (FBS), 2 mM L-glutamine, sodium pyruvate, 100 U/mL penicillin-streptomycin, and 10 mM HEPES. Cells were cultured at 37°C with 5% CO₂. Latently infected Jurkat cells were generated via infection with the HIV-GKO virus. HEK293T cells (ATCC, CRL-11268), used for transfection and viral production, were maintained in DMEM (Gibco) with 10% FBS and penicillin-streptomycin.

### Virus stocks

HIV-GKO viral stocks were produced by co-transfecting HEK293T cells (ATCC CRL-3216) with the HIV-GKO plasmid (Addgene #112234) and the VSV-G envelope plasmid pMD2.G (Addgene #12259) using Mirus-LT1 reagent (Mirus Bio, MIR2305) at a 1:4 VSV-G:HIV-GKO plasmid ratio. Transfected cells were cultured in DMEM (Gibco, 11995-065) with 10% FBS. At 48 h post-transfection, supernatants were centrifuged at 600 × *g* for 5 min and filtered through 0.45 μm membranes to remove debris and then aliquoted and stored at −80°C. Viral titers were determined by infecting Jurkat T cells (ATCC TIB-152), over a range of dilutions, followed by flow cytometry at 2 days post-infection and calculation of infectious units per mL. Jurkat cells were maintained in complete RPMI for up to 3 days post-infection.

### Primary CD4 T-cell HIV latency model

Total CD4 T cells from seronegative donors were activated for 72 h with anti-CD3/CD28 beads (Fisher Scientific) and then infected with a GFP-expressing reporter strain of HIV (HIV-Δ6-drEGFP-Thy1.2) (34) by spinoculation with viral supernatant at 600 g for 2 h at room temperature with 1 μg/mL polybrene. At 48 h post-infection, GFP^+^ cells were isolated by flow sorting using an Aria II flow sorter (Becton Dickinson) and then cultured for 2 weeks in the presence of 100 U/mL interleukin (IL)-2. After this period of culture, latently infected cells (GFP^−^) were enriched by a second round of flow sorting and expanded by additional stimulation with anti-CD3/CD28 beads for 2 weeks, before cryopreservation in liquid nitrogen. To examine the impact of latency reversing agents (LRAs) on these cells, vials of frozen cells were thawed, rested for 24 h in RPMI and 20 U/mL IL-2, before stimulation with LRAs for 24 h and flow cytometry.

### Small molecule inhibitor screen

A focused epigenetic inhibitor library consisting of 84 small molecules (assembled by the James Lab, UNC-CH) was initially dispensed at 10 mM in a 384-well format. Compounds were transferred to 96-well plates and diluted to 1 mM in DMSO and then further diluted to 200 µM in RPMI (1:5) to maintain final DMSO concentrations ≤ 0.1%. For screening, 1 µL of each 200 µM compound was added to V-bottom 96-well plates containing 30,000–50,000 HIV-GKO-infected Jurkat cells per well, 2 h post-infection, in a final volume of 200 µL. The layout was repeated across four technical replicate plates. DMSO vehicle controls were prepared using the same dilution scheme. After 3 days of concurrent infection and treatment, plates were centrifuged at 300 × *g* for 5 min, and cells were processed for flow cytometry.

### Flow cytometry

Viral gene expression was assessed by flow cytometry using mKO (HIV-GKO), eGFP (2D10), or the surface marker mouse heat-stable antigen (mHSA) (N6; BD Biosciences, 553262), depending on the model system. Cell viability was determined using Zombie Violet dye (BioLegend, 423113), diluted 1:1,000 in phosphate-buffered saline (PBS) (Thermo Scientific, 14190144). Cells were washed with PBS, stained with viability dye and relevant markers for 15 min, and then washed with 1 mL FACS buffer (49 mL PBS + 100 µL 100 mM EDTA + 1 mL heat-inactivated FBS), fixed in 4% paraformaldehyde (ChemCruz, sc-281692), and resuspended in 200 µL FACS buffer. Data were acquired on a BD Fortessa or Celesta instruments and analyzed using FlowJo v10.10.0. A minimum of 10,000 live (Zombie Violet-negative) events were collected per sample when possible.

### CRISPR/Cas9 gene knockouts

Protospacer targeting CRISPR RNA (crRNA) sequences for TAF1 and BRD1 were pre-designed by Integrated DNA Technologies (IDT) or Broad Institute CRISPick (41, 42), and the sequences for Tat (CCUUAGGCAUCUCCUAUGGC) and non-targeting controls (ACGGAGGCUAAGCGUCGCAA) came from previous literature (34). crRNAs were synthesized by Integrated DNA Technologies (IDT) (Coralville, IA, USA). CRISPR/Cas9 single-guide RNAs (sgRNAs) were made by mixing the crRNA and tracrRNA (IDT, 1072533) 1:1 at 100 µM, then annealing them in duplex buffer by heating to 95°C, and letting them cool gradually to room temperature using a thermocycler. Ribonucleoproteins (RNPs) were freshly assembled by combining 0.8 µL of Cas9 enzyme (IDT, 1081058), 0.8 µL of poly-glutamic acid (15 kDa, 100 mg/mL) (Alamanda Polymers, CAS# 26247-79-0), and 1 µL of the annealed sgRNA (100 pmol), at a final molar ratio of 2:1 RNA to Cas9. To target TAF1 and BRD1, we multiplexed three single-guide RNAs to improve knockout efficiency: TAF1 (1- UCACAGGGCACCGUCACGCG, 2- GGAGCGCCGGUACGUGCGC, 3- CGGUGUGGCCACUUAUCCUC) and BRD1 (1- GUGGAGCUCGCGGCGUAUCG, 2- GGGGAAUUAUUCGGCGCGUA, 3- GGGCUCGUCGUAUAGUAGCG). We pelleted 1 million cells per nucleofection of the 2D10 cell line (Generous gift from Dr. Jonathan Karn) (24) at 90 relative centrifugal force (RCF) for 10 min. Cells were washed with PBS and pelleted at 90 RCF for 10 min and then resuspended in 20 µL of Lonza SE nucleofection buffer (Lonza, V4SC-1096). Cells were added to the RNPs and nucleofected using the CL-120 program on the Lonza 4D system. Immediately after nucleofection, cells were transferred into pre-warmed RPMI, rested at 37°C for 10 min, and then resuspended at 400,000 cells/mL in complete RPMI (34). Viral gene expression was measured via changes in GFP by flow cytometry at 5 and 7 days post-nucleofection (DPN). At 5 and 7 DPN, we collected 1 million cells for Western blot to determine depletion efficacy. The LRAs used to stimulate TAF1-depleted cells were vorinostat (500 nM, SelleckChem, S1047), prostratin (125 nM, SigmaAldrich, P0077), or iBET-151 (125 nM, SelleckChem, S2780).

### Cell lysates and Western blotting

To generate protein lysates, approximately 1 million cells per sample were washed in PBS and lysed in RIPA buffer (Thermo Scientific, 89900) supplemented with complete protease inhibitors (Roche, 11697498001) and DNase I (Pierce, 88700) for 30 min at 4°C with gentle agitation. Lysates were centrifuged at 16,000 × *g* for 10 min to remove debris, and protein concentrations were quantified using the DC Protein Assay (Bio-Rad, 5000111). Equal amounts of protein (5–10 µg per sample) were resolved on a 4%–8% Tris-Acetate SDS-PAGE gel (ThermoFisher, EA03755BOX) and transferred onto a PVDF membrane (Invitrogen, IB23002). Membranes were blocked in Tris-buffered saline (TBS) with 5% milk, incubated overnight with primary antibodies, and washed in TBST (TBS + 0.1% Tween-20). The primary antibodies used were anti-TAF1 (ab264327), anti-BRD1 (EPR12960), and anti-beta-actin (ab49900) as a loading control. HRP-conjugated secondary antibodies (Novex, A16035) were applied, and signal was developed using SuperSignal West Pico PLUS chemiluminescent substrate (Thermo Scientific, 34577) and imaged with a ChemiDoc MP system (Bio-Rad, Universal Hood III).

### Bulk RNA-sequencing

RNA was extracted from non-targeting control and TAF1-depleted 2D10 cells using the RNeasy Plus Kit (Qiagen, 74134) and eluted in elution buffer. RNA concentration was measured with the Qubit RNA HS Assay (Invitrogen), and integrity was assessed using an RNA Nano Kit on a TapeStation 4150 (Agilent). Libraries were prepared using the Watchmaker RNA Library Prep Kit (Techtum, 7K0077-096), quantified by Qubit, and sequenced on an Illumina NextSeq 2000 with 2 × 50 bp paired-end reads. Transcript counts were generated from aligned reads using RNASTAR (v2.7.1a), and differential expression analysis was performed with DESeq2 (v1.44.0).

### CUT&RUN

CUT&RUN was performed using the CUTANA CUT&RUN Kit (EpiCypher, 14-1048) with minor modifications. 2D10 cells were nucleofected with CRISPR/Cas9 RNPs targeting TAF1 or non-targeting controls as described and collected in triplicate per antibody condition. For each replicate, 2 × 10⁶ cells were washed, bound to ConA-coated beads for 30 min at 4°C, and then divided into four groups and incubated overnight at 4°C in antibody buffer containing 0.01% digitonin and 0.5 µg of antibody. Antibodies included IgG (EpiCypher, 13-0042k), H3K4me3 (EpiCypher, 13-0060k), H3K9ac (Cell Signaling, 9649S), and RNA Polymerase II (Cell Signaling, 2629). DNA was quantified with the Qubit High Sensitivity dsDNA Assay (Invitrogen, Q328554).

Libraries were prepared using the CUTANA DNA Library Prep Kit (EpiCypher, 14-1001, Primer Set 1) with 14 cycles of PCR using dual-indexed barcodes. Libraries were quantified with Qubit and TapeStation 4150, pooled, and sequenced (150 bp paired-end, 20 million read pairs per sample) on an Illumina NextSeq 2000. Reads were demultiplexed with mkfastq, quality-checked with FastQC (v0.12.1), and trimmed with BBMap (v39.19). Alignment was performed using Bowtie2 (v2.4.5) to a custom genome combining GRCh38, HIV reference, and *Escherichia coli* K12 (as pseudo-chromosomes). BAM files were indexed and merged using Samtools (v1.21). BigWig files for visualization were generated with bamCoverage (DeepTools v3.5.4), and coverage across the HIV genome was quantified using bigWigAverageOverBed (UCSC-tools).

### Statistical analysis

To account for potential plate effects in the compound screen, we used the exact stratified Wilcoxon test. This non-parametric method ranks observations within each plate and combines ranks across plates, enabling group comparisons while controlling for intra-plate variability. For grouped comparisons across experimental conditions, one-way analysis of variance was applied. Dunnett’s multiple comparisons test was used when comparing multiple groups to a single control (e.g., non-targeting), while Tukey’s or Holm-Šidák corrections were used for all pairwise or selected group comparisons, as appropriate. These approaches provided robust statistical assessment across different replicate structures and experimental designs.

## Data Availability

All underlying data files are available at the UNC Dataverse: https://doi.org/10.15139/S3/ZAKZEV.

## SUPPLEMENTAL MATERIAL

Figure S1 mbio.01183-26-s0001.pdf BAY-299 toxicity and latency reversal dose curve.

Figure S2 mbio.01183-26-s0002.pdf Effect of BAY-299 on HIV latency and reactivation in primary CD4 T cells.

Figure S3 mbio.01183-26-s0003.pdf Effect of BAY-299 on HIV latency in the absence of TAF1 expression.

Figure S4 mbio.01183-26-s0004.pdf Principal component analysis of RNAseq data from TAF1 depleted cells.

Supplemental legends mbio.01183-26-s0005.docx Supplemental figure and table legends.

Table S1 mbio.01183-26-s0006.xlsx Detailed data from compound screen.

Table S2 mbio.01183-26-s0007.xlsx Bliss analysis of BAY-299 combination with latency reversing agents.

ASM does not own the copyrights to Supplemental Material that may be linked to, or accessed through, an article. The authors have granted ASM a non-exclusive, world-wide license to publish the Supplemental Material files. Please contact the corresponding author directly for reuse.
