# Supplementary figures and images for: A screen of chromatin-targeting compounds identifies TAF1 as a novel regulator of HIV latency
Source: mBio. 2026 Jun 15;17(7):e01183-26. doi: 10.1128/mbio.01183-26 (PMC13343944; doi:10.1128/mbio.01183-26)

A.

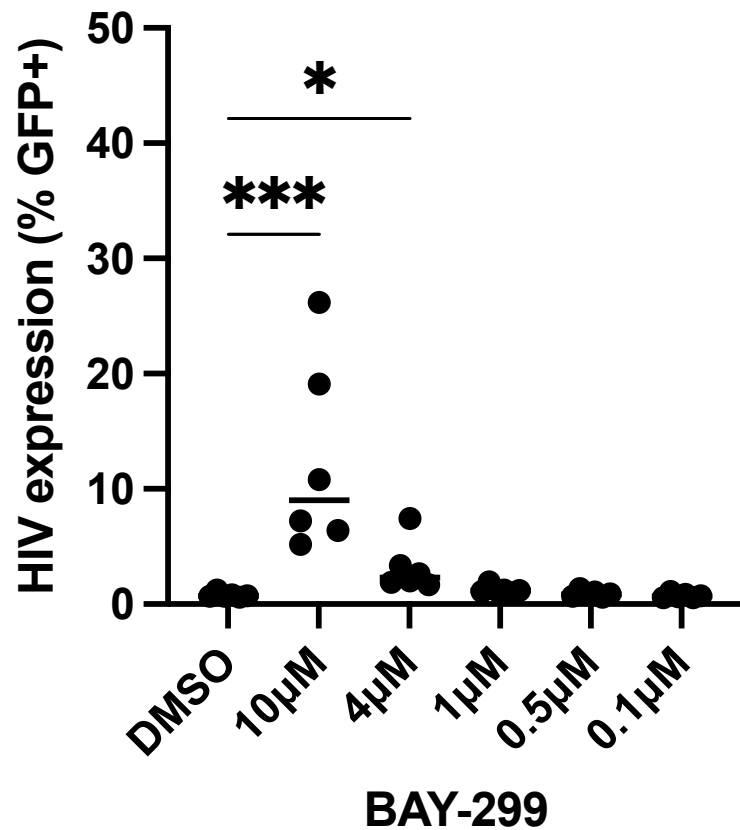

B.

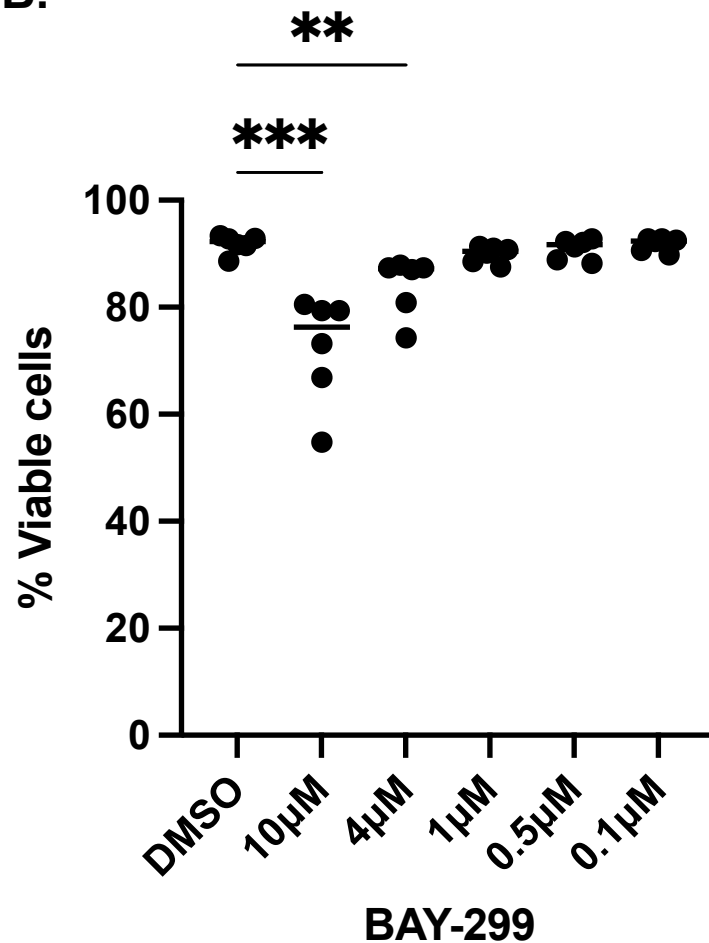

Supplement: Figure S1 — BAY-299 toxicity and latency reversal dose curve. [file mbio.01183-26-s0001.pdf]

**A.**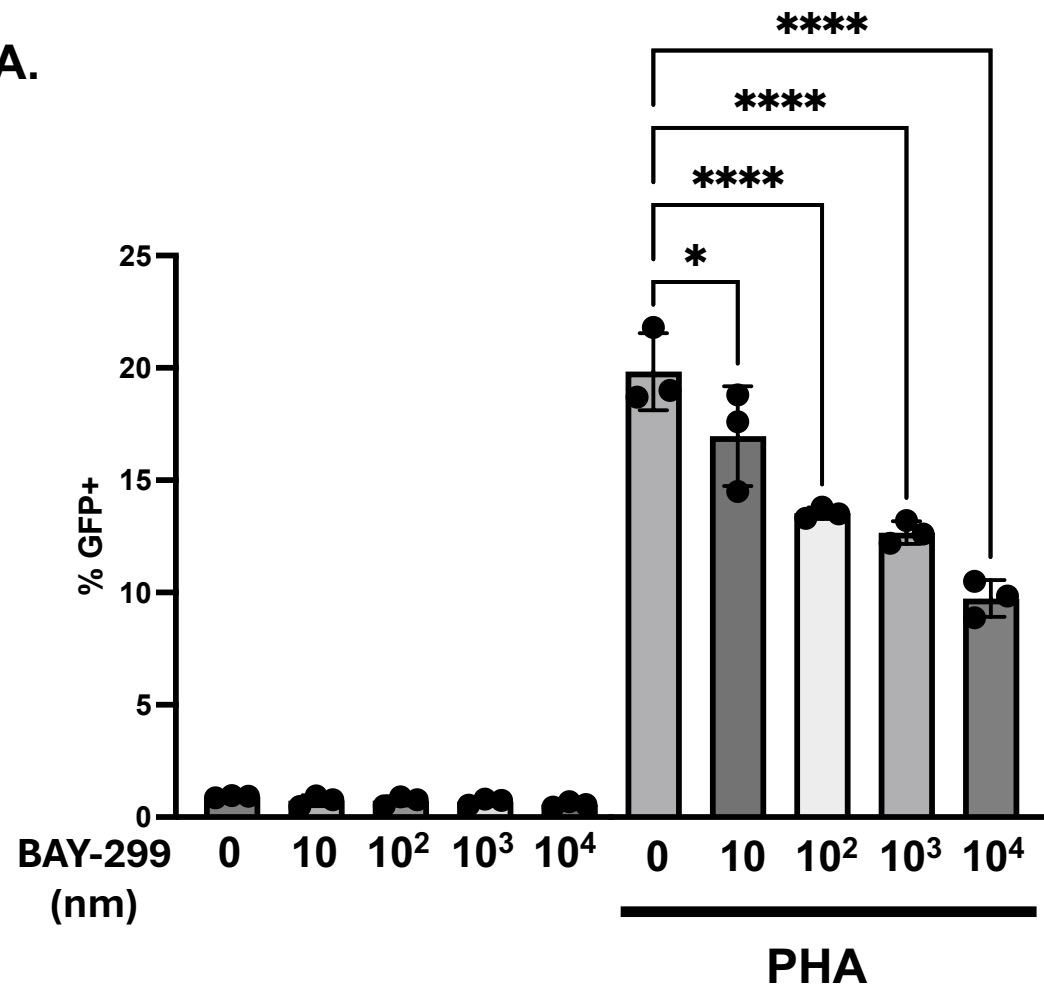**B.**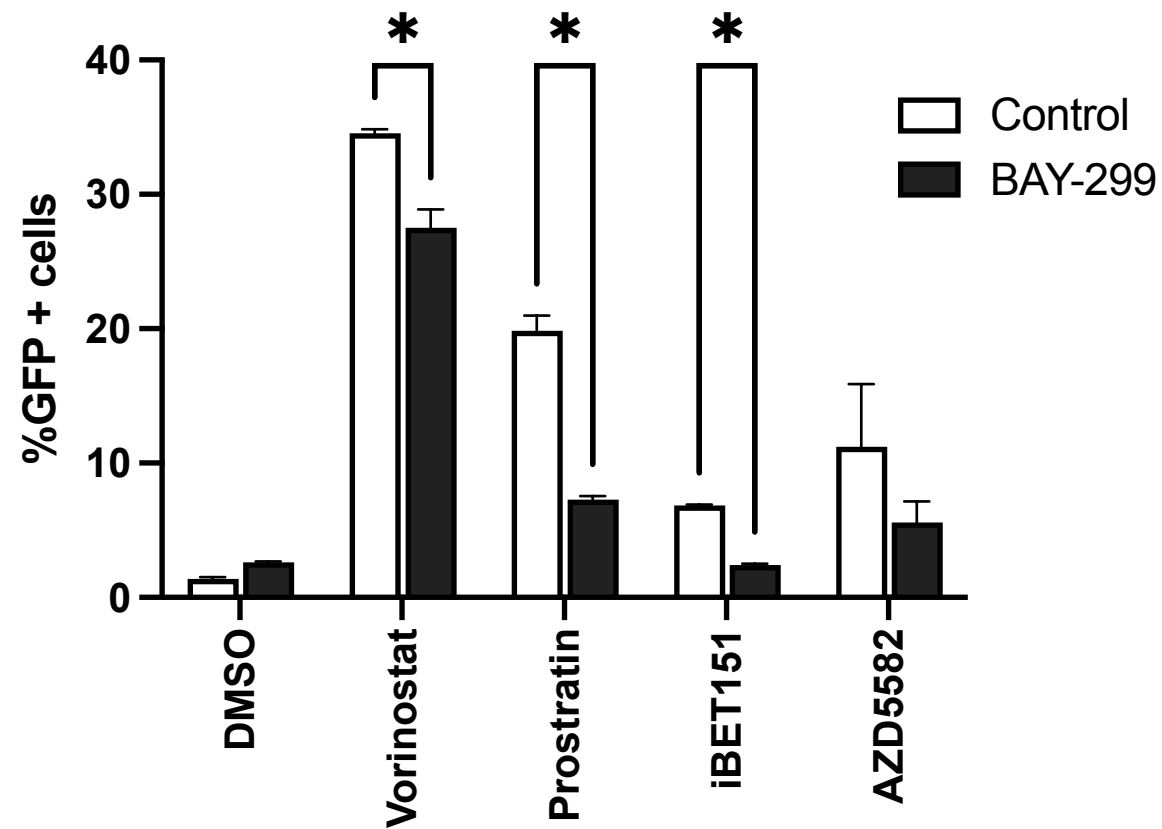

Supplement: Figure S2 — Effect of BAY-299 on HIV latency and reactivation in primary CD4 T cells. [file mbio.01183-26-s0002.pdf]

A.

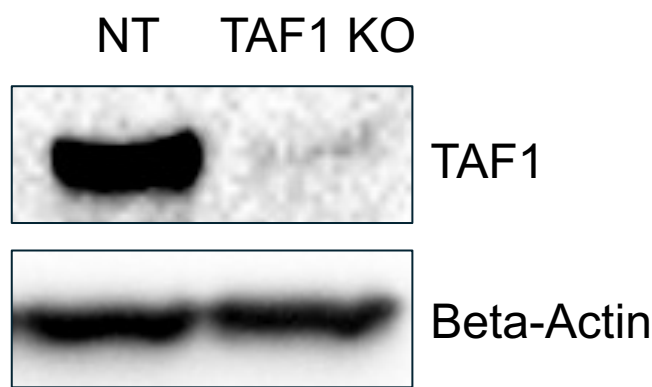

B.

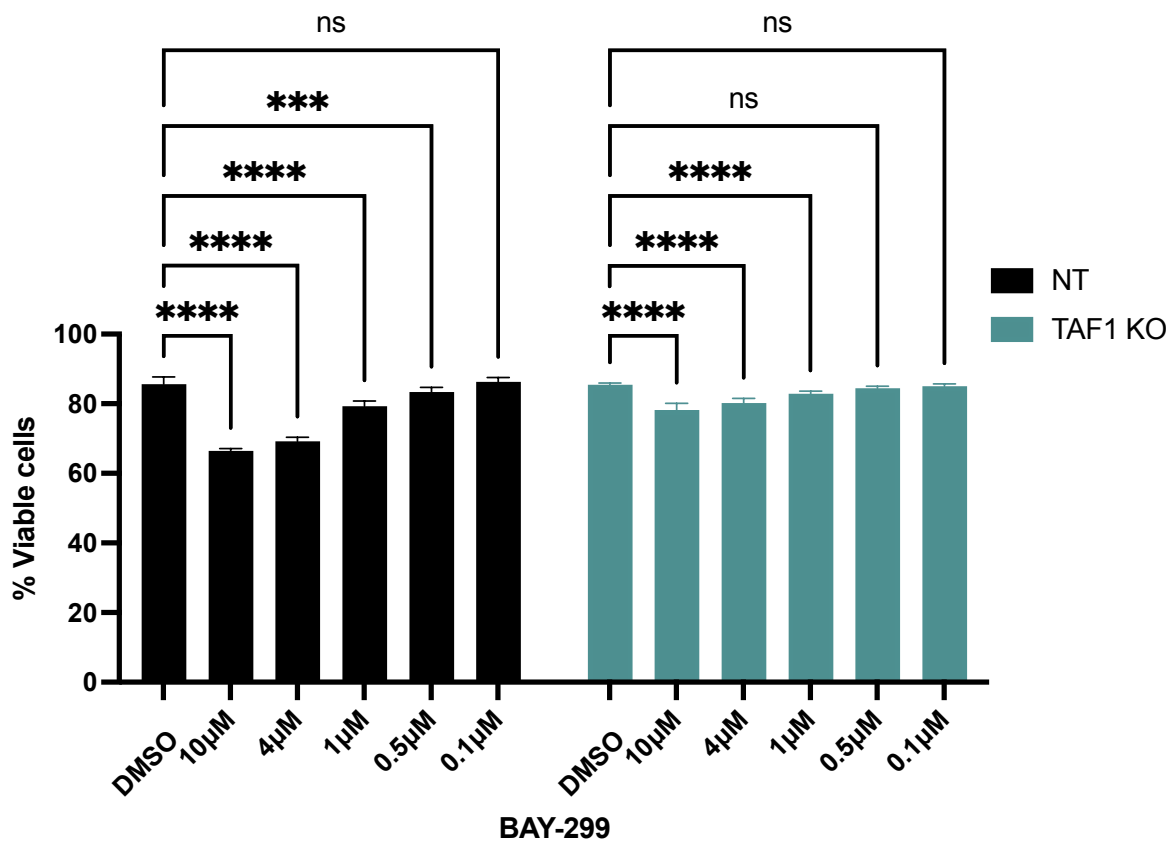

C.

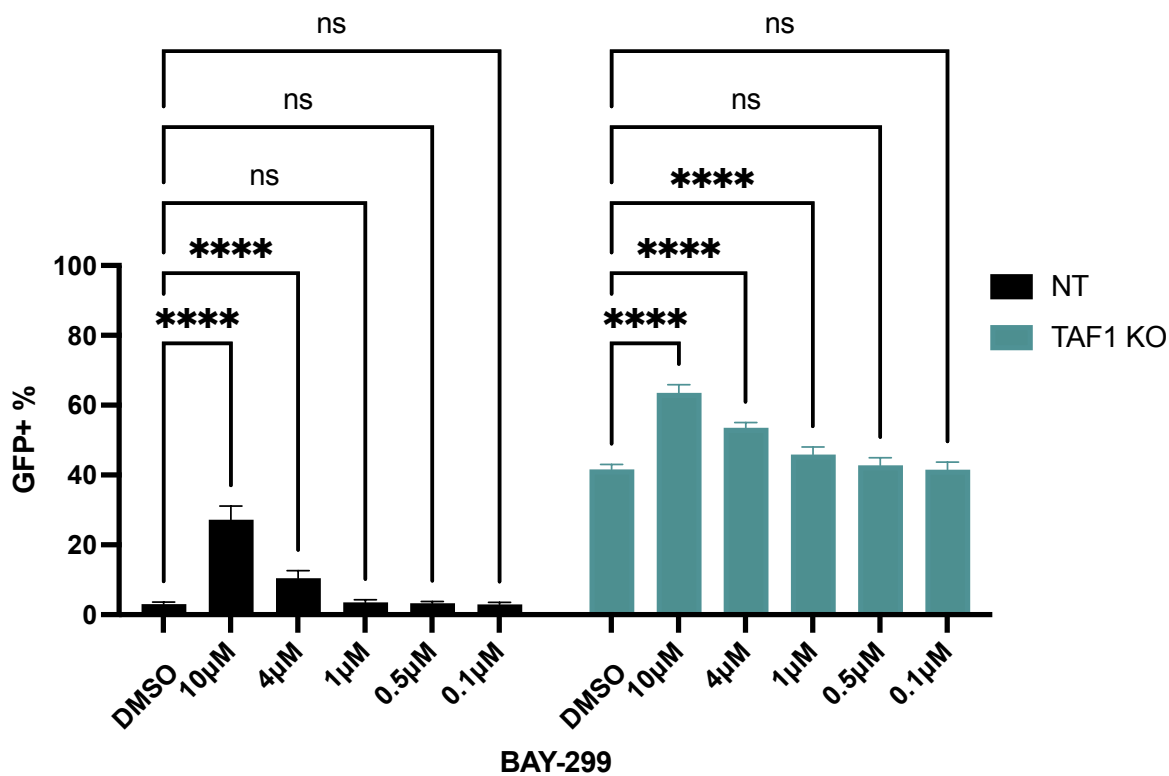

Supplement: Figure S3 — Effect of BAY-299 on HIV latency in the absence of TAF1 expression. [file mbio.01183-26-s0003.pdf]

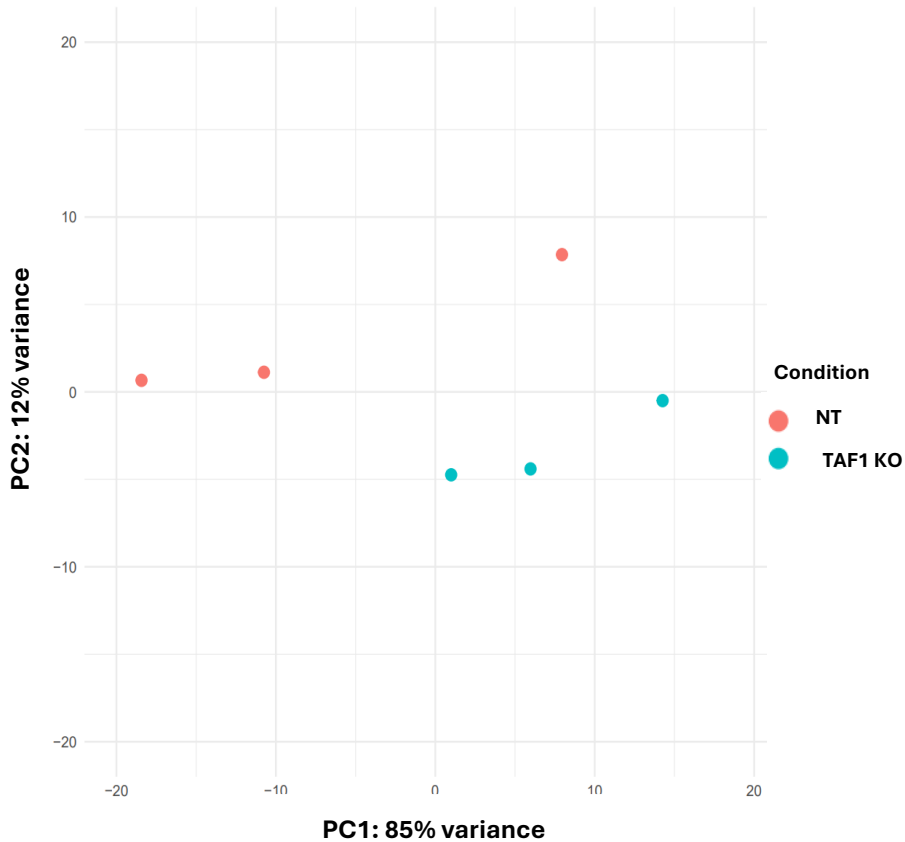

Supplement: Figure S4 — Principal component analysis of RNAseq data from TAF1 depleted cells. [file mbio.01183-26-s0004.pdf]
