# Supplementary material for: A screen of chromatin-targeting compounds identifies TAF1 as a novel regulator of HIV latency
Source: mBio. 2026 Jun 15;17(7):e01183-26. doi: 10.1128/mbio.01183-26 (PMC13343944; doi:10.1128/mbio.01183-26)
Supplement: Supplemental legends — Supplemental figure and table legends. [file mbio.01183-26-s0005.docx]

*Figure S1: BAY-299 toxicity and latency reversal dose curve.*

2D10 cells containing a latent copy of a GFP reporter virus were stimulated with BAY-299 at indicated concentrations for 24 h, followed by flow cytometry for (**A**) viral reactivation by percent GFP expression in the population, and (**B**) viability (using Zombie Violet staining). Each dot represents a single replicate. Asterices indicates statistical significance (Kruskal-Wallis test). *= P<0.05, **= P<0.01, ***=P<0.001

*Figure S2: Effect of BAY-299 on HIV latency and reactivation in primary CD4 T cells.*

**A**) Primary CD4 T cells that were latently infected with a GFP expressing strain of HIV (HIV-GFP) were stimulated with BAY-299 over a range of concentrations in the presence or absence of Phytohaemagglutinin (PHA) at 2.5μg/mL. At 24 h, viral reactivation was measured by flow cytometry. **B**) Primary CD4 T cells that were latently infected with a GFP expressing strain of HIV (HIV-GFP) were stimulated with BAY-299 at 10μM in the presence or absence of latency reversing agents vorinostat (500nM) prostratin (500nM), iBET151 (500nM) or AZD5582 (100nM). At 24 h, viral reactivation was measured by flow cytometry. Each bar represents the mean of triplicate readings. Asterices indicate statistical significance (**= P<0.05, **= P<0.01, ***=P<0.001).

*Figure S3: Effect of BAY-299 on HIV latency in the absence of TAF1 expression.*

2D10 cells were nucleofected with Cas9 ribonucleoprotein (RNP) particles with a non-targeting sgRNA (NT) or an sgRNA targeting TAF1. At 6 days post nucleofection, depletion of TAF1 was confirmed by western blot (**A**), and the cells were stimulated with a dose curve of BAY-299. At 24 h post stimulation, viability (**B**) and viral gene expression (**C**) (percent GFP+) was measured by flow cytometry. Each bar represents the average of 12 independent replicates. Asterices indicates statistical significance (Kruskal-Wallis test). *= P<0.05, **= P<0.01, ***=P<0.001.

*Figure S4: Principal component analysis of RNAseq data from TAF1 depleted cells.*

2D10 cells were nucleofected with Cas9 ribonucleoprotein (RNP) particles with a non-targeting sgRNA (NT) or an sgRNA targeting TAF1 in three independent cultures. At 6 days post nucleofection, RNA was isolated from the bulk population and analyzed by RNA sequencing (RNAseq). Plot shows Principal Component Analysis (PCA) of the transcriptomes from each sample, with non-targeting samples shown in red and TAF1-depleted samples in blue.

*Table S1: Detailed data from compound screen*

*Table S2: Bliss analysis of BAY-299 combination with latency reversing agents*

Data from Figure 2B was analyzed using a Bliss independence analysis. Positive Bliss scores (highlighted in green) indicate synergistic interaction, while negative scores indicate antagonistic interactions (highlighted in red).
